# The effect of nano-curcumin on HbA1c, fasting blood glucose, and lipid profile in diabetic subjects: a randomized clinical trial

**Published:** 2016

**Authors:** Hamid Reza Rahimi, Amir Hooshang Mohammadpour, Mostafa Dastani, Mahmoud Reza Jaafari, Khalil Abnous, Majid Ghayour Mobarhan, Reza Kazemi Oskuee

**Affiliations:** 1 *Student Research Committee, Department of Modern Sciences & Technologies, School of Medicine, Mashhad University of Medical Sciences, Mashhad, Iran*; 2 *Pharmacetical Research Center, School of Pharmacy, Mashhad University of Medical Sciences, Mashhad, Iran*; 3 *Cardiovascular Research Center, School of Medicine, Mashhad University of Medical Sciences, Mashhad, Iran*; 4 *Nanotechnology Research Center, School of Pharmacy, Mashhad University of Medical Science, Mashhad, Iran Biotechnology Research Center, School of Pharmacy, Mashhad University of Medical Science, Mashhad, Iran*; 5 *Pharmaceutical Research Center, Department of Medicinal Chemistry, Mashhad University of Medical Sciences, Mashhad, Iran*; 6 *Neurogenic Inflammation Research Center and Department of Medical Biotechnology, Mashhad University of Medical Sciences, Mashhad, Iran*

**Keywords:** *Curcumin*, *HbA1c*, *Fast blood glucose*, *Lipid profile*

## Abstract

**Objective:**

Diabetes mellitus is defined as a group of metabolic diseases characterized by hyperglycemia resulting from defects in insulin secretion, insulin action, or both or insulin resistance. Curcumin inhibits NF-κB signaling pathway. The aim of this study is evaluation of the effect of Nano-curcumin on HbA1C, fast blood glucose and lipid profile in diabetic patients.

**Materials and Methods:**

Seventy type-2 diabetic patients (fasting blood glucose (FBG) ≥ 126 mg/dL or 2-hr postprandial blood glucose ≥200 mg/dl) randomly receivedeither Curcumin (as nano-micelle 80 mg/day) or placebo for 3 months in a double blind randomized clinical trial. Fasting blood glucose, HbA1C, and lipids profile were checked before and after the intervention. Data analyses, including parametric and nonparametric tests were done using the SPSS 11.5 software. A p value < 0.05 was regarded as statistically significant. (RCT registration code: IRCT2013081114330N1)

**Results:**

Mean age, BMI, FBG, total cholesterol (TC), triglyceride (TG), LDL, HDL, HbA1c , and sex and had no significant difference at the baseline between the groups. In Nano-curcumin group, a significant decrease was found in HbA1C, FBG, TG, and BMI comparing results of each subject before and after the treatment (p<0.05).

By comparing pre- and post-treatment values among the groups, HbA1c, eAG, LDL-C, and BMI variables showed significant differences (p<0.05).

**Conclusion:**

These findings suggest an HbA1c lowering effect for Nano-curcumin in type-2 diabetes; also, it is partially decrease in serum LDL-C and BMI.

## Introduction

Diabetes mellitus is defined as a group of metabolic diseases characterized by hyperglycemia resulting from defects in insulin secretion, insulin action, or both or insulin resistance in skeletal muscle, liver and adipose tissues with a failure of b-cell compensation and a relative insulin deficiency (Association 2013 ▶; Pettitt et al. 2014 ▶). 

The prevalence of diabetes globally was estimated to be 2.8% in 2000 and 4.4% in 2030 and is higher in men than women (Wild et al. 2004 ▶). There are 23.6 million children and adults in the United States, or 7.8% of the population in the USA who have diabetes. While an estimated number of 17.9 million have been diagnosed with diabetes, unfortunately, 5.7 million people (nearly one-quarter) are unaware that they have the disease. The Iranian diabetic population is estimated to be more than 1.5 million (Larejani and Zahedi 2001 ▶).

**Figure 1:**
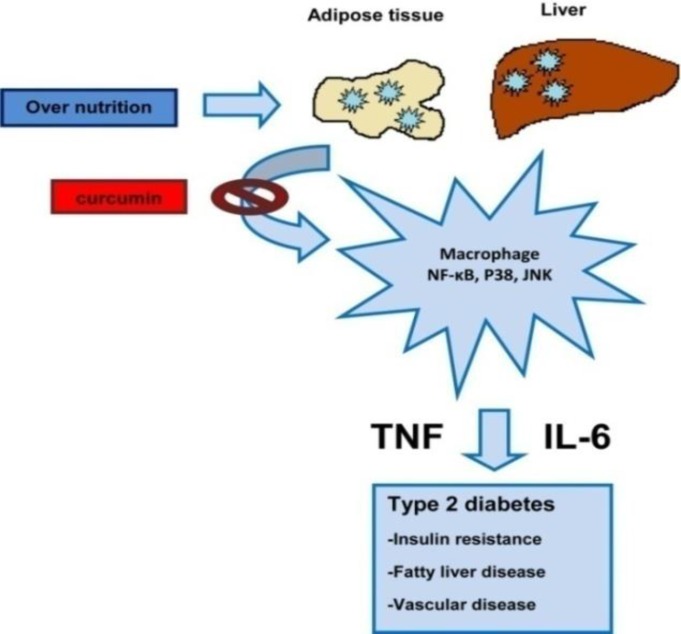
Over-nutrition could influence on cytokine production by macrophages, liver, and adipose tissue. These cytokines produce insulin resistance and fatty liver(JNK: Jun N-terminal kinase, NF-ĸB: nuclear factor kappa-light-chain-enhancer of activated B cells) (Gao and Bataller 2011

Free fatty acid metabolites could activate the serine kinase pathway. This signaling pathway influences insulin signaling and insulin receptor functions (Figure 1)(Saini 2010 ▶).

Curcumin is a very active component which comes from the root of turmeric (in Farsi it is called Zardchobeh) (Chuengsamarn et al. 2014 ▶; Rahimi and Kazemi Oskuee 2014 ▶).

The chemical name of curcumin is diferuloylmethane or (1E, 6E)-1, 7-bis (4-hydroxy-3-methoxyphenyl)- 1, 6-heptadiene-3, 5-dione (Hatcher et al. 2008 ▶; Santel et al. 2008 ▶). Curcumin has antioxidative, anti-inflammatory, chemopreventive, and chemotherapeutic activity(Menon and Sudheer 2007 ▶; Sandur et al. 2007 ▶) with no significant side effects (Rahimi and Kazemi Oskuee 2014 ▶) it can also regulate multiple cell signaling pathways(Gupta et al. 2013 ▶). 

Nuclear factor-κB (NF-κB) signaling pathway is one of the most important pathways in the cellular and molecular mechanisms of inflammation. In this cellular signaling pathway, cytokines and adhesion molecules are secreted. According to the molecular studies, curcumin inhibits NF-κB signaling pathway, which could regulate cytokines production and influence the immune response. Curcumin suppresses some genes expression, especially cytokines genes. Curcumin could down-regulate the expression of TNF α, IL-1, IL-6, IL-8, adhesion molecules (ICAM, VCAM), C-reactive protein (Rahimi and Kazemi Oskuee 2014 ▶; Rahimi et al. 2015 ▶).

Curcumin can have a therapeutic effect onall human chronic diseases such as asthma, bronchitis, inflammatory bowel disease, rheumatoid arthritis, coronary artery disease, atherosclerosis plaque stabilizing, diabetes mellitus, obesity, fatty liver, metabolic syndrome, depression, cancer, and allergy (Aggarwal and Harikumar 2009 ▶).

Curcumin administration in the pre-diabetic population decreased the number of pre-diabetic individuals who eventually developed type-2 diabetes mellitus properly (T2DM) (Chuengsamarn et al. 2012 ▶).

**Nano-curcumin**

Nano-Curcumin is a registered curcumin product (SinaCurcumin®) for oral use which has been developed in Nanotechnology Research Center of Mashhad University of Medical Sciences, Mashhad, Iran and marketed by Exir Nano Sina Company in Tehran-Iran (IRC:1228225765). Each soft gel of Nano-curcumin contains 80 mg of curcumin in the form of nano-micelle. The oral absorption of curcumin is very poor due to its hydrophobic nature. However, Nano-curcumin has a significantly higher bioavailability after oral use compared to the simple powder of curcumin (Rahimi, Jaafari et al. 2015 ▶). 

In the present study, we examined the effect of Nano micelle curcumin on HbA1C, fasting blood glucose and lipid profile in diabetic subjectsin comparison with the placebo group.

## Materials and Methods

**Study design and participants**

A double blind randomized placebo-control add-on clinical trial (registration code: IRCT2013081114330N1) was set up in Mashhad University of medical Sciences.

Seventy type-2 diabetic patients (fasting blood glucose (FBG) ≥ 126 mg/dl or 2-hr postprandial blood glucose ≥200 mg/dl) were randomly assigned to Nano-curcumin (as nano-micelle 80 mg/day) or placebo for 3 months in a double blind randomized clinical trial. According to the ethical issues all other necessary medications were given to subjects, so, this study is an add-on therapy. All patients were observed by the same researcher during this study. 

**Randomization procedures**

**Figure 2:**
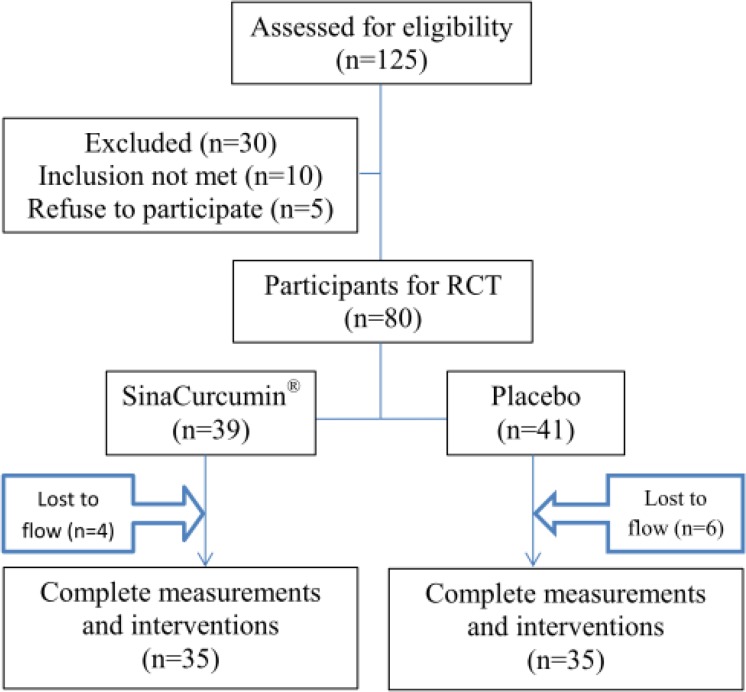
Flow chart of participants

After screening process, diet and lifestyle training were done face-to-face. All subjects were randomly assigned to either the Nano-curcumin (as nano-micelle 80 mg/day) or placebo-treated group (control condition) using a fixed randomization scheme based on random numbers provided by a computer software. The subjects were informed that two methods of interventions were being evaluated (Figure 2).

**Inclusion criteria**

Subjectswho were included in the present study were:

Suspected CAD by the selected cardiologist, Male/ Female older than 18 years of age, and they understood the study procedures and agreed to participate.

**Exclusion criteria for case and control group**

Pregnant and breast-feeding patients were excluded from the present study. In addition, based on the case and control subject’s past medical history taken by the cardiologist, those who reported any of the following diseases were also excluded from the study:Rheumatic disease, chronic liver disease, renal disease, any type of cancer, infectious disease in the last 3 months, people with any surgical procedures in the preceding 3 months, subjects who had a history of angioplasty or coronary by-pass graft surgery, subjects who had a traumatic or major immunologic disease, subjects who were usingsteroids, penicillin, oral contraceptive or hormone replacement therapy. Also,subjects with any inflammatory disease such as inflammatory bowel disease, psoriasis, multiple sclerosis, systemic lupus erythematosus, myasthenia gravis, autoimmune thyroiditis were excluded from the study (de Freitas, Pinheiro, Miranda, Thiers, de Barros Vieira, Pernambuco and Pe 2001; Kazemi-Bajestani, Ghayour-Mobarhan, Ebrahimi, Moohebati, Esmaeili and Ferns 2008; MJ Zibaee Nezhad, P Ghanbari, B Shahryari and Aghasadeghi 2009).

**Collecting subjects' data**

A general questionnaire was used to obtain information including demographic data and anthropometric parameters. According to the standard procedures, blood pressure was measured using a mercury sphygmomanometer and periodical validity and reliability of the instrument was checked. Height was measured using a wall-mounted stadiometer. Weight was measured using electronic scales while the subject was wearing light clothing without shoes.

**Laboratory tests**

Blood samples were obtained in the early morning after an overnight fasting. Fasting blood samples (10 ml) were collected into plain Vacutainer™ tubes, for lipid profile, HbA1C.For measurement of fasting blood glucose, blood was taken into Vacutainer™ tubes containing fluoride-oxalate (Kazemi-Bajestani, Ghayour-Mobarhan, Ebrahimi, Moohebati, Esmaeili and Ferns 2007 ▶).

Blood samples were centrifuged for plasma separation (4000 RPM, 4 min). Total cholesterol, low density lipoprotein cholesterol, high density lipoprotein, cholesterol, and glucose were measured using routine techniques by a Cobas auto-analyser system (ABX Diagnostics, Montpellier, France) (Kazemi-Bajestani et al. 2007 ▶).

The results of the fasting blood sugar (FBS) < 110 mg/dl were interpreted using the American Diabetic Association criteria: normal values between 110 and 126 mg/dl, and those > 126 mg/dl are considered as impaired fasting glucose (IFG) and DM, respectivelyall(Pekkanen e al. 1999 ▶; Kazemi-Bajestani et al. 2007 ▶).

**Ethical issues**

All participants were provided with information about the study both verbally and by written informed consent. All those who hadthe exclusion criteria including those who preferred not to attend at any stage were withdrawn from the study. Each patient gave informed written consent to participate in the study, which was approved by the Ethics Committee of Mashhad University of Medical Sciences. This form was agreed and completed by all subjects. The investigation conforms to the principles outlined in the Declaration of Helsinki.

**Sample Size**

Sample size was calculated using frequency data of previous studies (Chuengsamarn, Rattanamongkolgul, Phonrat, Tungtrongchitr and Jirawatnotai 2014 ▶; Ghorbani, Hekmatdoost and Mirmiran 2014 ▶; Nauck, Meininger, Sheng, Terranella and Stein 2007 ▶) with an 80% power, 5% level of significance, and a SD of 35.3. We enrolled at least 35 subjects in each treatment group.

**Statistical analysis**

All statistical analyses were performed using SPSS for (of) Windows™, version 11.5 software package (SPSS Inc., Chicago, IL, USA). At first, quantitative data were assessed using Kolmogorov-Smirnov tests to check normality. Data were expressed as Mean ± SD for parameters with a normal distribution or median and interquartile range for non-normally distributed data. Group comparisons were performed using sample T-Test or Mann-Whitney U test (in case of non-normally distributed data). A two-sided p value < 0.05 was considered statistically significant.

## Results

Baseline demographic and biochemical serum parameters are matched (such as age, sex, body mass index, smoking habit, hypertension, fasting blood glucose, HbA1c, estimated Average Glucose (eAG), total cholesterol, low density lipoprotein cholesterol, high density lipoprotein cholesterol, and triglyceride) theyshowed) no statistically significant difference between the groups(Tables 1 and 2).

**Table 1:** Baseline characteristics of the study population stratified by groups

**Variables**		**Groups**		**p value**
		**Nano-curcumin** **N=35**	**Placebo** **N=35**	
**Age(y)**		56.34 ± 11.17	60.95 ± 10.77	0.132
**Sex**	Male	17(48.5)	14(40)	0.518
	Female	18(51.5)	21(60)	
**BMI (kg/m** ^2^ **)**	Normal	3(8.5)	5(14.3)	>0.999
	Overweight	26(74.2)	25(71.4)	
	Obesity	6(17.3)	5(14.3)	
**Smoking habit**	Current	4(11.5)	3(8.6)	0.949
	Former	10(28.5)	9(25.7)	
	Never	21(60)	23(65.7)	
**Hypertension, No.** (**%)**	Yes	18(51.5)	21(60)	0.518
	No	17(48.5)	14(40)	

BMI: Body Mass Index. Data are shown as Mean±SD or No.(%).

**Table 2:** Serum biochemical parameters and some other characteristics of study groups after clinical trial

**Variables**		**Before**		**After**		**p value**			
		**Groups**		**Groups**					
		**Nano-curcumin** **N=35**	**Placebo** **N=35**	**Nano-curcumin** **N=35**	**Placebo** **N=35**	**P1**	**P2**	**P3**	**P4**
**FBG(mg/dl)**		135.5±51.33	148.30±76.41	120.29±38.01	176.0±61.56	0.154	0.004	0.049	0.204
**HbA1c (%)**		7.59±1.74	7.49±1.75	7.31±1.54	9.00±2.33	0.838	0.02	<0.001	0.521
**eAG**		171.2±50.0	168.4±50.35	167.00±51.0	211.6±66.9	0.838	0.050	<0.001	0.521
**TC (mg/dl)**		163.4±33.94	162.4±38.59	158.62±44.06	149.00±24.62	>0.999	0.542	0.018	0.245
**LDL-C (mg/dl)**		96.57±33.94	99.78±30.33	91.04±28.72	84.00±12.59	0.723	0.486	0.01	0.316
**HDL-C (mg/dl)**		54.30±14.02	60.35±15.96	60.95±15.68	55.00±11.09	0.064	0.734	0.014	0.323
**TG (mg/dl)(median(IQR))**		109(94.75)	142(97.50)	131(60.27)	113(58.0)	0.475	0.050	<0.001	0.387
**BMI(kg/m** ^2^ **)**		26.92±2.71	27.27±3.59	25.57±2.71	27.50±3.38	0.667	0.019	<0.001	0.452
**BMI (kg/m** ^2^ **)**	**Normal**	3(8.5)	5(14.3)	12(34)	5(14.3)	>0.999	0.042	0.032	0.705
	**Overweight**	26(74.2)	25(71.4)	22(63)	25(71.4)				
	**Obesity**	6(17.3)	5(14.3)	1(3)	5(14.3)				

BMI: Body Mass Index, FBG: Fasting blood glucose, TC: total cholesterol, HDL-C: High density lipoprotein- cholesterol, LDL-C: Low density lipoprotein- cholesterol, TG: triglyceride, IQR: Interquartile Range. Data are shown as Mean±SD or median (IQR) or No.(%).eAG: estimated Average Glucose =(28.7×HbA1CHbA1c)-46.7.

P1 is the p value of comparing Nano-curcumin with Placebo before intervention.

P2 is the p value of comparing Nano-curcumin with Placebo after intervention.

P3 is the p value of comparing data in Nano-curcumin group between before and after using pair T test.

P4 is the p value of comparing data in Placebo group between before and after using pair T test.

In Table 2, body mass index, fasting blood glucose, HbA1C, estimated Average Glucose (eAG), total cholesterol, low density lipoprotein cholesterol, high density lipoprotein cholesterol, and triglyceride were compared between the 2 groups after the intervention.Curcumin improved FBG, HbA1c, BMI, and eAG, but had no effect on LDL, HDL, TG and TC(Table 2).

**Figure 3:**
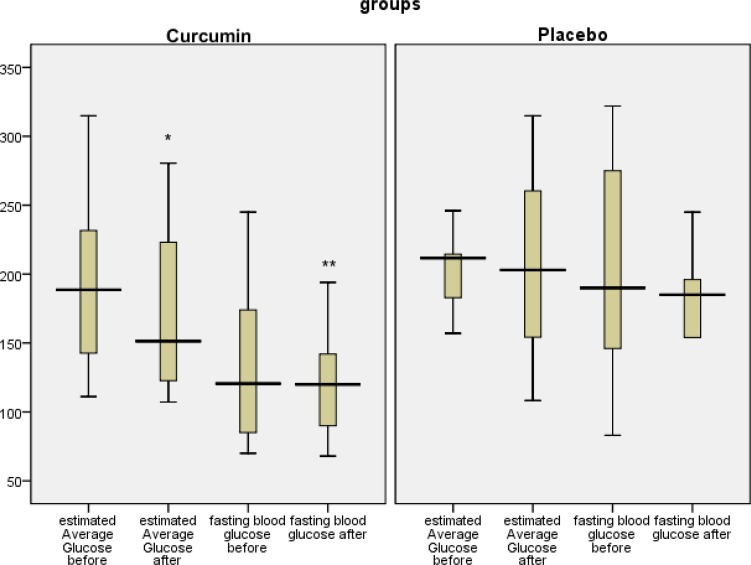
Fasting blood glucose and estimated Average Glucose, before and after the intervention in both groups. *p<0.05, **p<0.01: statistically significant difference between before and after the intervention using pair t test

**Figure 4:**
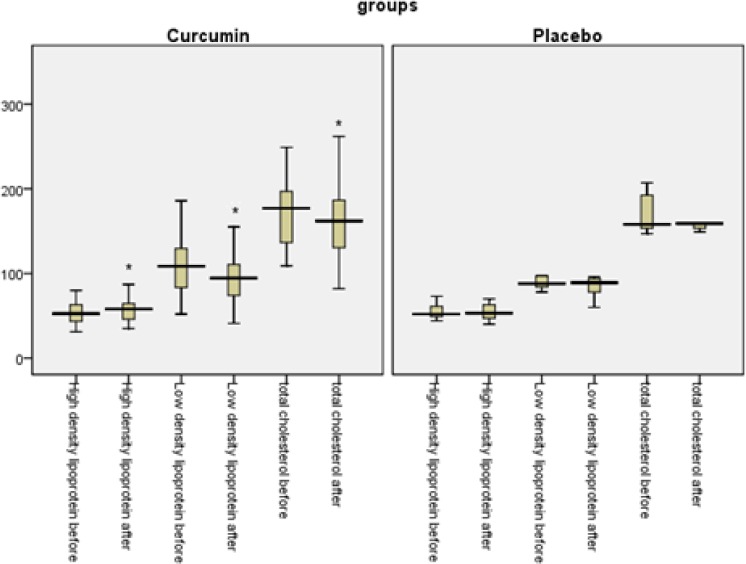
Total cholesterol, High-density lipoprotein-cholesterol, Low-density lipoprotein- cholesterol before and after intervention in both groups. *Statistically significant difference between before and after the intervention (P<0.05) using pair and independent T test

Pairedt test was used to compare FBG, HbA1c, eAG, TG, TC, LDL-C, and HDL-C for each subject before and after the intervention. Regarding all above-mentioned variables, in curcumin-treated group, statistically significant differences were found before and after the intervention (p=0.049, p<0.001, p<0.001, p=0.018, p=0.01, p=0.014, and p<0.001, respectively). However, in placebo group, there were no differences between the results of FBG, HbA1c, eAG, TG, TC, LDL-C, and HDL-C in baseline and endpoint results (p=0.204, p=0.521, p=0.521, p=0.254, p=0.316, p=0.323, and p=0.387)(Table 2 and 3 and Figures 3 and 4).

Determination of longitudinal changes of FBG, HbA1c, eAG, TG, TC, LDL-C, HDL-C, and BMI before and after of this is shown in Table 3. Significant difference was found between groups in HbA1c, eAG, LDL-C, and BMI (p<0.05) (Table 3).

**Table 3:** Determination (delta, Δ) of changes in some variables before and after the study

**Variables**	**Nano-curcumin** **N=35** **(Δ=after- before)**	**Placebo** **N=35** **(Δ=after- before)**	**p value**
**ΔFBG**	-17.12±40.38	-24.88±53.94	0.657
**ΔHbA1c**	-0.91±1.11	0.33±1.48	0.013
**ΔeAG**	-21.88±25.42	9.56±42.71	0.015
**ΔTC**	-15.45±44.75	-13.11±36.74	0.889
**ΔLDL-C**	-16.41±30.93	-8.95±25.51	0.046
**ΔHDL-C**	1.83±13.11	2.11±6.91	0.952
**ΔTG**	-6.7±67.52	-15.33±37.39	0.772
**ΔBMI**	-1.34±1.88	0.22±1.31	0.001

Δ=after- before for ech value, BMI: Body Mass Index, FBG: Fasting blood glucose, TC: total cholesterol, HDL-C: High density lipoprotein- cholesterol, LDL-C: Low density lipoprotein- cholesterol, TG: triglyceride, IQR: Interquartile Range.eAG: estimated Average Glucose =(28.7×HbA1CHbA1c)-46.7

As noted before (Figure 2), 10 patients in both groups did not complete the procedure of this study, due to long distance to the institution, their health insurance and crowdedness of Ghaem Academic hospital, Mashhad, Iran.

## Discussion

A large number of studies has been conducted on the physicochemical traits and pharmacological effects of curcumin on different diseases like cardiovascular diseases, diabetes, cancer, rheumatoid arthritis, Alzheimer’s, inflammatory bowel disease (IBD), or even wound healing, due to its minor toxicity (Soni and Kuttan 1992 ▶; Garodia et al. 2007 ▶; Liu et al. 2013 ▶). The use of curcumin for treatment of these conditions has revealed that curcumin modulates several transcription factors, kinases, growth factors, cytokines, and some other enzymes (Kakarala et al. 2010 ▶; Thamake et al. 2011 ▶; Venkatesha et al. 2011 ▶). Other studies showed that curcumin has anti-diabetic activities (Yeh et al. 2003 ▶; Hsu and Cheng 2007 ▶).

Insulin resistance (IR) is the backbone of T2DM (Na et al. 2011 ▶); It was reported that curcumin could break IR (Neerati et al. 2014). Also, curcumin could increase the activation of PPAR-γ (Nishiyama et al. 2005 ▶). PPAR-γ activity suppresses low-density lipoprotein (LDL) receptor and it could be used as a treatment of hypercholesterolemia (Kang and Chen 2009 ▶).

Curcumin may prevent diabetes mellitus microvascular and macrovascular complications (Sharma et al. 2006 ▶; Muthenna et al. 2009 ▶). 

A great problem that should be considered is that several studies on chemical structure of curcumin (a polyphenolic compound) have shown that this substance has a poor bioavailability (Chainani-Wu 2003 ▶; Anand et al. 2007 ▶; Hatcher, Planalp et al. 2008 ▶). In addition, it has a low absorption, fast metabolism, and fast systemic elimination (Anand, Kunnumakkara et al. 2007 ▶).

To overcome curcumin's poor bioavailability, nano-micelle containing curcumin which is a registered curcumin product (SinaCurcumin®) was prepared for oral use. These nano-micelles are prepared from GRAS (generally recognized as safe) pharmaceutical excipients and C3-complex form of curcumin. The percentage of encapsulation of curcumin in this nano-micelle is close to 100% and their sizes are around 10 nm. Nano-curcumin has a significantly higher bioavailability after oral use as compared to simple powder of curcumin due to the following reasons:An intact layer of water is on the surface of intestinal epithelial cells (unstirred water layer), so any medication should pass this barrier (Smithson et al. 1981 ▶), this is a confronting barrier for lipophilic molecules such as curcumin. Bile salts largely facilitates absorption and soluble substances such as lipophilic vitamins, lipids, fatty acids and cholesterol(Howles 2010 ▶) and also the same happens for curcumin as nano-micelles. After oral administration, soft gel of Nano-curcumin opens in the stomach in less than 15 minutes and will be diffused to the small intestine; this product has a higher bioavailability than other similar products(Rahimi and Kazemi Oskuee 2014 ▶). 

In this clinical trial, we found that Nano-curcumin reduces FBG and HbA1c during 3 months of therapy; it could also significantly reduce eAG and lipid profile parameters. Obesity is one of the most important factors in IR and T2DM development (Weisberg et al. 2008 ▶). In this research, we found that curcumin has a good influence on BMI in diabetic patients. 

Also, it was found that one-month oral administration of curcumin (1 gram/day) could reduce triglycerides concentrations in obese subjects (Mohammadi et al. 2013 ▶).

According to the Framingham Heart Study, level of HbA1c has a great association with cardiovascular disease and T2DM (Singer et al. 1992 ▶).

Moreover, it was shown that low dose of oral curcumin could reduce LDL-C and TC (Alwi et al. 2008 ▶). We compared serum levels of TC, TG, LDL-C, and HDL-C, before and after the treatment and significant differences were found for each subject in Nano-curcumin group.

## Note

*Please cite this paper as*

Rahimi HR, Mohammadpour AH, Dastani M, Jafari MR, Abnous K, Mobarhan MG, Kazemi Oskuee R.The effect of nano-curcumin on HbA1c, fasting blood glucose, and lipid profile in diabetic subjects: a randomized clinical trial.Avicenna J Phytomed, 2016; 6 (5): 567-577.
